# Trajectories of re‐engagement: factors and mechanisms enabling patient return to HIV care in Zambia

**DOI:** 10.1002/jia2.26067

**Published:** 2023-02-24

**Authors:** Laura K. Beres, Chanda Mwamba, Carolyn Bolton‐Moore, Caitlin E. Kennedy, Sandra Simbeza, Stephanie M. Topp, Kombatende Sikombe, Njekwa Mukamba, Aaloke Mody, Sheree R. Schwartz, Elvin Geng, Charles B. Holmes, Izukanji Sikazwe, Julie A. Denison

**Affiliations:** ^1^ Department of International Health Johns Hopkins Bloomberg School of Public Health Baltimore Maryland USA; ^2^ Centre for Infectious Disease Research in Zambia Lusaka Zambia; ^3^ Department of Infectious Diseases University of Alabama at Birmingham Birmingham Alabama USA; ^4^ College of Public Health Medical and Veterinary Sciences James Cook University Townsville Queensland Australia; ^5^ Department of Public Health Environments and Society Faculty of Public Health and Policy, London School of Hygiene and Tropical Medicine London UK; ^6^ University of Washington St. Louis St. Louis Missouri USA; ^7^ Department of Epidemiology Johns Hopkins Bloomberg School of Public Health Baltimore Maryland USA; ^8^ Georgetown University Washington DC USA

**Keywords:** adherence, Africa, health systems, HIV care continuum, retention, social support

## Abstract

**Introduction:**

While disengagement from HIV care threatens the health of persons living with HIV (PLWH) and incidence‐reduction targets, re‐engagement is a critical step towards positive outcomes. Studies that establish a deeper understanding of successful return to clinical care among previously disengaged PLWH and the factors supporting re‐engagement are essential to facilitate long‐term care continuity.

**Methods:**

We conducted narrative, patient‐centred, in‐depth interviews between January and June 2019 with 20 PLWH in Lusaka, Zambia, who had disengaged and then re‐engaged in HIV care, identified through electronic medical records (EMRs). We applied narrative analysis techniques, and deductive and inductive thematic analysis to identify engagement patterns and enablers of return.

**Results:**

We inductively identified five trajectories of care engagement, suggesting patterns in patient characteristics, experienced barriers and return facilitators that may aid intervention targeting including: (1) intermittent engagement;(2) mostly engaged; (3) delayed linkage after testing; (4) needs time to initiate antiretroviral therapy (ART); and (5) re‐engagement with ART initiation. Patient‐identified periods of disengagement from care did not always align with care gaps indicated in the EMR. Key, interactive re‐engagement facilitators experienced by participants, with varied importance across trajectories, included a desire for physical wellness and social support manifested through verbal encouragement, facility outreach or personal facility connections and family instrumental support. The mechanisms through which facilitators led to return were: (1) the promising of living out one's life priorities; (2) feeling valued; (3) fostering interpersonal accountability; (4) re‐entry navigation support; (5) facilitated care and treatment access; and (6) management of significant barriers, such as depression.

**Conclusions:**

While preliminary, the identified trajectories may guide interventions to support re‐engagement, such as offering flexible ART access to patients with intermittent engagement patterns instead of stable patients only. Further, for re‐engagement interventions to achieve impact, they must activate mechanisms underlying re‐engagement behaviours. For example, facility outreach that reminds a patient to return to care but does not affirm a patient's value or navigate re‐entry is unlikely to be effective. The demonstrated importance of positive health facility connections reinforces a growing call for patient‐centred care. Additionally, interventions should consider the important role communities play in fostering treatment motivation and overcoming practical barriers.

## 1 INTRODUCTION

Returning to HIV care after disengagement is a common but poorly understood health behaviour [1] that supports long‐term health and reduces HIV transmission [2, 3, 4, 5]. Re‐engagement is increasingly recognized as part of the HIV care cascade, where individuals move in and out of care following diagnosis [6, 7, 8, 9, 10] and across a lifetime [1]. Studies estimate 25–75% of disengaged patients return to care across 6 months to 15 years of follow‐up, with higher return closer to initial disengagement [6, 11, 12, 13].

While factors driving disengagement are well‐studied [11, 14, 15, 16, 17, 18], little is known about facilitating return [19]. Research in North America and Europe found that physical and mental health [12, 20, 21, 22], stability including housing permanence and lack of substance use [12, 22], social support and positive relationships with care providers [20, 22, 23] were associated with return. Re‐engagement studies in East Africa are mainly quantitative identifying female gender, travel‐related missed visits and absence of treatment fatigue as associated with return [11, 24]. Effective re‐engagement support requires understanding both patients’ experiences and how their interpretations of their experiences translate into returning to care [25, 26]. Only three qualitative studies from sub‐Saharan Africa have investigated re‐engagement. They identified social support [27, 28, 29], combined with the motivation to remain healthy [28, 29] and health worker reminders [29], as critical to return.

Additional research is needed to understand facilitators of and patient perspectives on re‐engagement. We conducted the first qualitative study of re‐engagement in Zambia, using a patient‐centred approach to understand factors influencing re‐engagement and the mechanisms through which they operated.

## 2 METHODS

### 2.1 Study background

We recruited participants from a quantitative HIV care outcomes study among a representative, probability‐based sample of 2769 adult Zambian clients who were “lost to follow‐up” [30]. Patients had at least one HIV care visit between August 2013 and July 2015 and subsequent >90‐day visit gap in their electronic medical record (EMR) [30, 31]. From September 2015 to July 2016, peer educators tracked these patients in four provinces, locating 603 who self‐identified as disengaged from care. These patients were surveyed on their care experiences and encouraged to return [30, 32]. Quantitative analysis of follow‐up EMR data identified re‐engaged patients and factors associated with return (Table S1) [13]. We selected our qualitative study sample from re‐engaged parent study participants in Lusaka Province (*N* = 96).

### 2.2 Approach

Informed by constructivist epistemology [33] and a narrative research approach [34], we conducted in‐depth interviews to gather HIV care experience narratives. Symbolic interactionism [35] guided our focus on how patients constructed meaning from their experiences.

### 2.3 Sampling and recruitment

**Figure 1:**
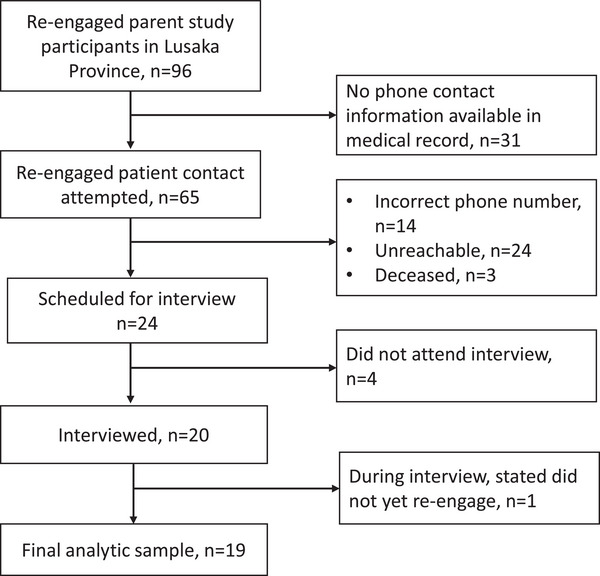
Recruitment outcomes.

Our qualitative sampling initially sought variation in gender and patient care engagement status (currently engaged in care vs. repeatedly disengaged). The interviewer (CM) conducted rolling recruitment with outcomes shown in Figure 1, assessing sample variation with the lead qualitative researcher (LB). Using ongoing data review, LB and CM determined that saturation was reached on facilitators, our primary area of interest, and ended recruitment after 20 participants.

**Figure 2:**
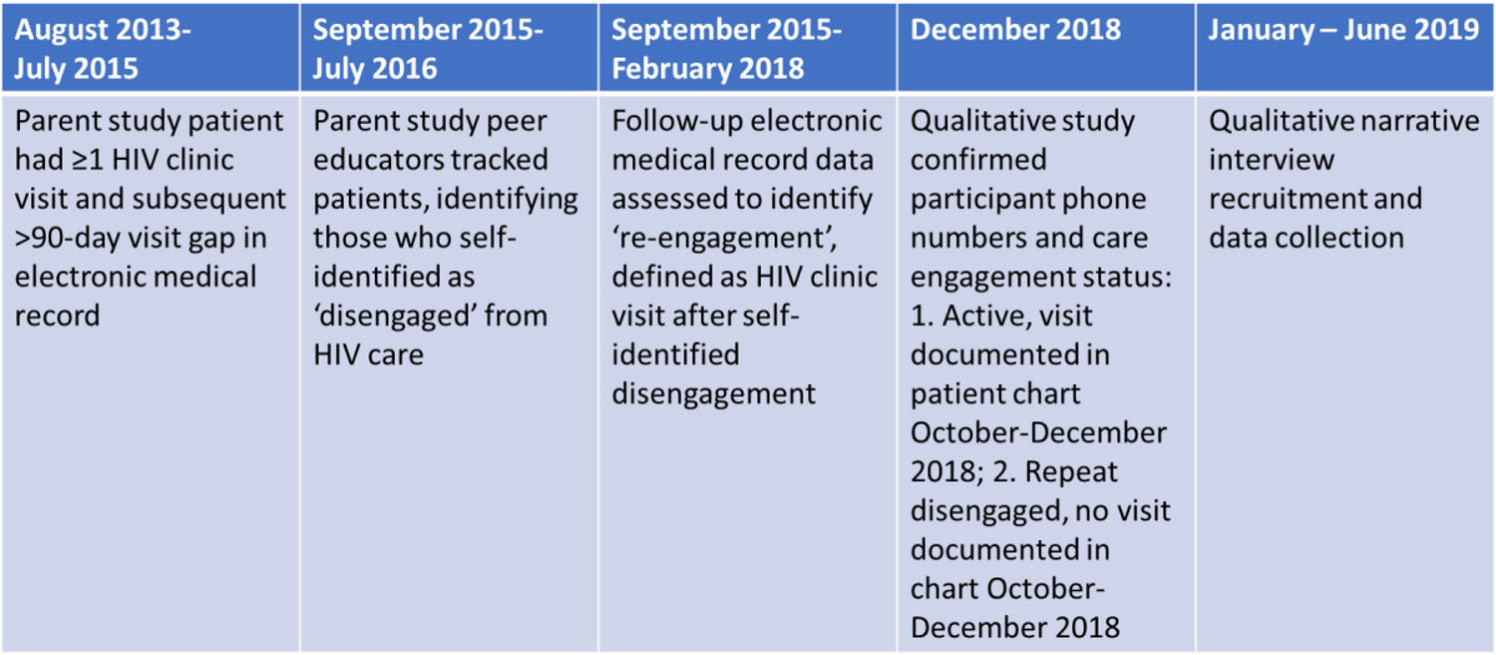
Parent and qualitative study timeline.

Between January and June 2019, we conducted 22 interviews, 1–2.5 hours long, with 20 participants from 10 health facilities (Figure 2). Five interviewees spoke Bemba, eight Nyanja and seven English. The sample included 13 females, seven males and six patients who had repeat disengagement. All participants began HIV care during the period when CD4 count guided antiretroviral therapy (ART) initiation. Seven had initiated ART before disengagement (Table S1). During the narrative analysis, we excluded one participant from the results who, despite EMR‐documented re‐engagement, demonstrated she had not re‐engaged.

### 2.4 Data collection

We conducted narrative interviews to understand participant HIV care‐seeking journeys from the initial diagnosis to the present. Following a semi‐structured guide, we first elicited the participant's open‐ended HIV care narrative, and then probed to understand the people, experiences, contexts and events proximal to episodes of care disengagement and re‐engagement. We specifically inquired about re‐engagement‐associated factors from our previous quantitative analyses [13]. To prompt participants’ memories, we prepared an individualized guide for each participant, including EMR visit data and their parent study survey responses. If a participant did not recognize EMR dates or survey responses, we ceased that line of questioning. Interview questions were revised during data collection to explore new themes and improve question clarity. Initially planning two interviews per participant; after experiencing follow‐up challenges with the first two participants, we subsequently conducted interviews in a single session.

We conducted interviews in publicly accessible but private locations. Participants gave voluntary, written informed consent and were reimbursed ZMW 100 (∼USD 7.50) for transport.

### 2.5 Analysis

Interviews were transcribed verbatim, with simultaneous English translation. In two cases where audio recording permission was not granted, we typed interview notes. We uploaded transcripts, notes and analytic memos written within 24 hours of each interview into Nvivo 12 (QSR International, 2018) for analysis.

We utilized narrative analysis, iteratively reading, coding, categorizing, synthesizing and comparing the data to identify central themes. For each participant, we produced (1) a summary of care engagement events and important engagement influences and (2) a chronological narrative summary or “re‐storying” [36, 37] of the patient's care journey recounted from diagnosis to present, which often included multiple periods of disengagement and re‐engagement. Absent participant labelling of disengagement or re‐engagement periods themselves, we defined disengagement as a recounted absence from expected clinic visits or ART adherence, and re‐engagement as seeking health services after an absence.

We deductively coded transcripts and memos for factors significantly associated with re‐engagement in our prior quantitative work. We inductively identified descriptive and *in vivo* [38] codes for re‐engagement barriers, facilitators and experiences. We reviewed inductive codes after one‐third of the interviews were coded, then re‐read and re‐coded previously coded transcripts. We conducted additional rounds of inductive coding, matrix‐based coding comparisons and categorization of segments by facilitators of return [39]. We compared the chronological narrative summaries, inductively identifying common patterns of an episodic engagement or “care trajectories.” We examined participant‐level attributes and assessed the prominence of facilitators to establish within‐trajectory themes. Finally, we analysed the meaning and operation of facilitators to understand the mechanisms through which facilitators led to return. CM and LB sought consensus on all analyses through discussion, resolving disagreements through dialogue.

### 2.6 Reflexivity

CM conducted all recruitment, interviews and supported analysis. LB led study design and analysis. Before data collection, CM and LB wrote positionality memos, reflecting on their relationship to the study. CM, a Black, Zambian, female social scientist, fluent in English, Nyanja and Bemba, the three study languages, had extensive qualitative HIV‐related research experience. LB, a White, American female Ph.D. candidate, lived in Zambia 4 years and in southern Africa an additional 5 years working in mixed methods HIV research. CM and LB had worked together for several years, including as parent study co‐investigators. To foster reflexivity, CM and LB reviewed the quantitative findings on factors influencing return and postulated how they might influence care engagement. We included reflection on researcher positionality in analysis memos and discussions.

### 2.7 Ethical review

This study was approved by the University of Zambia Research Ethics Committee, the Zambian Ministry of Health and the University of Alabama at Birmingham Institutional Review Board.

## 3 RESULTS

### 3.1 Patient‐reported re‐engagement

All participants described periods of absence from, and return to, HIV care or treatment. However, their self‐perceived periods of disengagement often did not align with the gaps indicated in the EMR. More than half of the participants said their EMR did not accurately record their clinic visits. Several asserted that paper‐based clinic files were more accurate than EMRs. Patient perceptions of disengaged periods were heavily influenced by ART adherence. Several participants did not count missed appointments or extended absences from the clinic as disengagement because they had not yet started taking ART. Among those on ART, several said they accessed ART outside of traditional clinic visits, so they remained engaged in care without attending scheduled appointments. A few participants stated the EMR missed episodes of disengagement when it recorded successful clinic visits, but patients decided to not take ARVs at home.

### 3.2 Engagement trajectories

**Figure 3:**
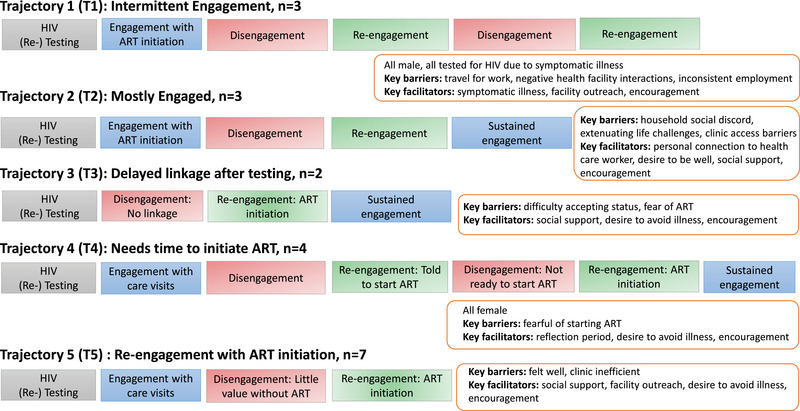
Trajectories of HIV care engagement.

Through analysis of participant‐narrated care interactions from the time of their HIV diagnosis to the interview, we identified five distinct patterns across time or “trajectories” (Figure 3) of patient‐identified engagement, disengagement and re‐engagement with HIV services:

1. Intermittent engagement,
2. Mostly engaged,
3. Delayed linkage after testing,
4. Needs time to initiate ART and
5. Re‐engagement with ART initiation.

These trajectories demonstrate important emerging patterns of re‐engagement facilitators, barriers and patient characteristics that differ by trajectory. Below, we present each trajectory. After the trajectories, we discuss facilitators more broadly.

*Trajectory 1: Intermittent engagement*, represents multiple cycles of disengagement and re‐engagement. Participants in T1 (*n* = 3) were all males who initially tested for HIV due to symptomatic illness, experienced sustained engagement barriers related to chronic work instability and returned to care due to repeat symptomatic illness. The main driver of disengagement was inflexible ART access that did not accommodate their livelihoods. They had difficulty attending clinic visits during work hours as strong competition for jobs meant any absence reduced earnings, perceived dependability and the likelihood of future employment. Additionally, employment often required unplanned travel for indeterminant durations. Their resulting need for flexibility in clinic attendance and/or the amount of ART distributed often engendered negative interactions with health workers, including scolding, accusations of selfishness and refusal to provide extended drug supply. The most prominent driver of re‐engagement in T1, participants returned to the clinic when they experienced a non‐resolving symptomatic illness that limited their daily activities. While receiving treatment for acute symptoms, they would also re‐start ART. In all T1 narratives, re‐engagement came from symptomatic illness combined with encouragement from family members and at least one episode of healthcare worker outreach. Encouragement reinforced that HIV must be dealt with or that symptomatic illness required attention thus persuading return to care, despite other priorities or conflicted feelings based on negative past experiences with health workers. Episodes of re‐engagement were sustained by patients negotiating sufficient ART supplies through drug‐sharing with other patients, drug pick‐up by patient supporters and clinic provision of informal, short‐term drug supplies during periods of travel.

*Trajectory 2: Mostly engaged*, includes participants who were generally in care with one key period of disengagement followed by re‐engagement. The three T2 participants discussed family support for initially seeking HIV treatment. Drivers of care interruptions varied, including household discord, treatment fatigue and clinic access barriers. However, they described similar facilitators of re‐engagement after an absence. Prominently, these facilitators included a desire to avoid illness and facility outreach, with each detailing an important personal connection at the facility. These connections, a family member or someone the participant developed a relationship with through facility interactions, offered confidential support for living with HIV, direct encouragement to re‐start, increased comfort in returning to the facility and improved treatment access (e.g. shorter wait times and home delivery). Personal connections helped to sustain T2 engagement after return through ongoing ease of treatment access and support to overcome periods of doubt or depressive thoughts.

*Trajectory 3: Delayed linkage after testing*, represents participants who disengaged after testing HIV positive. The two T3 participants initially rejected their HIV diagnosis and feared ART, including concern that starting ART and then missing doses would cause death. These barriers were exacerbated by a lack of evident value of care seeking, linked, for one participant, to being asymptomatic (male participant), and, for another, to fear of being blamed for bringing HIV into their relationship (female participant). Their re‐engagement was facilitated by socially supported re‐testing: the male participant's wife suggested couples testing when he became symptomatically ill, while the female participant and her husband were encouraged by a health worker family member to get couples’ testing. This family support helped sustain engagement by convincing them to begin ART, supporting rapid movement through the facility, assuring anonymity and physically accompanying them to the clinic.

*Trajectory 4: Needs time to initiate ART*, represents participants who engaged in care until it was recommended that they initiate ART (*n* = 4). All females, some described a fear of ART they needed to overcome, while others needed reflection and preparation before beginning a lifelong commitment to treatment. For each, return was facilitated by concern for their health combined with social support to re‐engage, including dialogue helping to overcome their resistance to ART. They discussed recognizing ART as improving their ability to live out their life priorities. In several cases, health workers supported delayed ART initiation by “giving time”; participants experienced this positively as supportive power‐sharing engendered eventual return.

*Trajectory 5: Re‐engagement with ART*, represents participants who sought HIV care after testing positive, disengaged from care due to lack of perceived value, and then re‐engaged to initiate ART. In T5 (*n* = 7) narratives, many were asymptomatic and felt clinic visits were inefficient and not worth their time and resources without ART. Facility outreach was the most prominent facilitator of re‐engagement in T5, with outreach directly offering or indirectly signalling an opportunity to access ART. Facility outreach, other social support and a desire to avoid illness furthered re‐engagement through underlining the promise of living out their life priorities associated with ART. Sustained engagement post‐return was supported by encouragement from loved ones and a desire to remain healthy.

### 3.3 Facilitators and mechanisms of return

In no case was re‐engagement enabled through a single facilitator. Most facilitators were interactive and operated across trajectories, with varying levels of prominence. Consistent with our approach, we conceptualize and describe facilitators of re‐engagement as *what* the participant experienced that supported return (i.e. the motivations, actors and actions they encountered). These represent potential interventions that facilities, families or others may implement or leverage to support re‐engagement. We also identified six underlying mechanisms through which facilitators translated to re‐engagement behaviour. These mechanisms, summarized below, describe *how* the facilitators resulted in re‐engagement (i.e. key characteristics interventional facilitators would need to be effective).

**Table 1:** Mechanisms underlying re‐engagement in care

Underlying mechanisms through which re‐engagement is supported	Types of barriers prominently overcome	Linked experiential facilitator(s)
Having the ability to live out one's life priorities	Involved in overcoming all barriers	A desire for physical wellbeing; instrumental family support
Feeling valued	Foundational in overcoming all barriers	Social support for re‐engagement: verbal encouragement, family, facility outreach, personal facility connections
Interpersonal accountability	Psychosocial (e.g. stigma and treatment fatigue) and clinic‐level access barriers	Social support for re‐engagement: verbal encouragement, family, facility outreach, personal facility connections
Re‐entry navigation support	Psychosocial (e.g. stigma and fear of health worker reprimand) and clinic‐level access barriers	Social support for re‐engagement: facility outreach, personal facility connections
Improved care or treatment access	Structural (e.g. conflict between work demands and treatment access options) and clinic‐level barriers	Social support for re‐engagement: facility outreach, personal facility connections
Supported negotiation of a patient‐specific barrier to return (e.g. depression and stigma)	Psychosocial barriers	Social support for re‐engagement: family, facility outreach, personal facility connections

#### 3.3.1 A desire for physical wellbeing

A desire for physical wellbeing was central to re‐engagement across all five trajectories, ranging from seeking care in response to symptomatic ill health (e.g. T1) to preserving wellbeing (e.g. T5). Physical wellness signified the ability to live a “normal” life, which included working, supporting family and avoiding stigma by not manifesting visible HIV symptoms. However, a desire for physical health was insufficient in isolation; it interacted with verbal encouragement, instrumental support, such as being accompanied to a facility, outreach to negotiate stigma and improved access to result in return.

#### 3.3.2 Social support for re‐engagement

Social support for re‐engagement was the other central facilitator across the five trajectories, with participants recounting the importance of different types of support (e.g. emotional, instrumental, informational and appraisal [40]) coming from different sources.

*Verbal encouragement* Importantly, all 19 participants from all five trajectories narrated that verbal encouragement offering emotional, informational and/or appraisal support facilitated their re‐engagement. Encouragement came from someone they loved or respected, including family members, friends, clinic employees, acquaintances living openly with HIV and clergy. A single episode of encouragement rarely enabled return. In their narratives, participants discussed experiencing, and needing, repeated encouragement, especially from multiple sources over time. Across trajectories, encouragement was variously discussed as (1) increasing self‐worth and motivation for self‐care; (2) fostering hope that physical wellness, work, supporting their family and enjoying life is possible with treatment; and (3) normalizing HIV, fostering self‐acceptance or eliminating the fear of rejection. These experiences reinforced an ability to live their lives according to their priorities. One man explained,

I was actually touched, my sister encouraged me [saying], “You just go. Where you see me right now [feeling healthy], I do take the drugs [ART]” and then I said “okay.”‐T5, 25–34 years.

The relationship with the person giving encouragement mattered more than what the person said. Kind words were ineffective from someone disliked or untrusted; for example, one woman, angry that her husband concealed his HIV‐positive status, ignored his encouragement. Similarly, words from unfriendly health workers intended to encourage re‐engagement were experienced as discouraging. However, when coming from trusted loved ones or caring health workers, multiple participants mentioned that even challenging words would encourage return by facilitating personal reflection, reinforcing the benefits of care, and, ultimately, conveying that participants were valued. One woman said her re‐engagement was facilitated by “the love around that people show you.” She continued: “Like my dad…he makes me understand that ‘it's not about you, it's also about us’…That actually brightens us up inside. Like, okay, this person cares.”‐T2, 18–24 years

*Facility‐based outreach and connections* Direct interactions with health facility‐employees featured as important experiences facilitating re‐engagement. With prominence in T2: Mostly engaged, and T5: Re‐engagement with ART initiation, participants described “facility outreach,” that is a health worker calling or visiting to explicitly encourage return after an extended absence as a key facilitator of re‐engagement. Facility outreach was mentioned throughout narratives across trajectories, as all participants had been contacted by a parent‐study‐employed peer educator tracer and encouraged to return. However, not all narratives linked outreach to re‐engagement. Among T5 participants, facility outreach alerted them to the opportunity to initiate ART, while among T2 participants, the exceptional circumstance that led to their disengagement from care was mitigated by outreach and an invitation to return. Participants generally did not specify in their narrative if the outreach was from a study‐employed or facility‐employed health worker. One participant said outreach moved him to act on a longstanding desire to return that he had not prioritized. However, most participants discussed more nuanced mechanisms leading to return that acted through facility outreach.

The patient's perception of whether the outreach worker cared about and respected them mediated the effect of outreach. One woman said:

I think also that the approach matters. If a person just calls you and then they just tell you that you need to come to the hospital, you would get scared to say, what have I done? Or has my disease…become AIDS now?…They approached me in a very friendly way, so it was so encouraging to go back, but if they ask you questions like “Why don't you come?”…that is kind of scary.‐T1, 18–24 years

Several participants for whom facility outreach did not lead to re‐engagement said health workers were just doing their jobs. Outreach did not make them feel special, although several acknowledged it *could* if approached in a meaningful way. Additionally, outreach was ineffective if participants did not perceive themselves as disengaged. For example, a T4 participant aged 25–34 years described having “been given” 6 months to accept ART initiation before starting treatment, and the outreach happened during these 6 months.

Additionally, prominent in T2 and T3, participants described having a personal connection to someone at the facility as key to their re‐engagement. These personal connections included family members who were health workers, health workers with whom they had previously established positive rapport, and in some cases, relationships that developed following facility outreach. Connections spanned facility roles, from medical doctors to cleaners. These interactions and connections translated to re‐engagement behaviours in several ways.

- *Feeling valued*: Multiple participants said that experiencing facility outreach communicated they were valued, and the facility was invested in their health. One woman said:
  Sometimes when you stop, and they call you to come and start medication. “Why did you stop?” and they tell you and advise you to continue taking treatment, and they show you love, love and care; they don't want you to be in a situation where you are sick or get back to being sick. They will encourage you, and you will feel loved. ‐T5, 25–34 years old

In most cases, facility outreach interacted with symptomatic illness or encouragement from family members to facilitate return. For one intermittently engaged man (T1), 25–34 years, an outreach worker telephoned a family member designated as his ART supporter. The family member then actively encouraged the participant to re‐engage, which allowed him to overcome his fear of being scolded and return.

(b) *Interpersonal accountability*: For some participants, a health worker's investment of time, energy, kindness and resources through facility outreach inspired a sense of improved self‐worth and motivation. Participants wanted to return that investment and demonstrate shared responsibility by re‐engaging. One woman said:
  I felt good. It felt like they were worried about me, worried about my health, so that is what made me follow their instructions. ‐T5, 35–44 years
(c) *Supported navigation of re‐entry*: Both facility outreach and having a personal connection at the facility offered navigation support. Participants whose re‐engagement was facilitated by outreach noted value in being given instructions on clinic day/date, whom to see and were assured a warm welcome. Conversely, one participant who experienced facility outreach but said it did not lead to his re‐engagement reported it lacked specific guidance on re‐entry. Similarly, multiple disengaged participants with a personal facility connection who considered returning to care called their connection in advance or sought the employee immediately upon presentation at the clinic to ensure a friendly interaction, avoid reprimand and identify the practical steps required to re‐engage. One woman who was nervous to return contacted a counsellor who had helped her navigate a serious non‐HIV‐related health concern several years prior.
  He told me: ‘You come early, you do this, you get your medicine, then you leave.’…he actually also counselled me…he helped me. ‐T4, 18–24 years
(d) *Improved care or treatment access*: Facility outreach and personal facility connections led to assurances that participants could start ART, with particular relevance to T5: Re‐engagement with ART initiation. They also improved the flexibility of ART access and scheduling appointments to accommodate work demands, which was important for re‐engagement in T1: Intermittent engagement and T3: Delayed linkage after testing—as well as for sustaining engagement in T2: Mostly engaged. Privileged access through a personal connection included obtaining ART without a facility visit, skipping the queue or moving through it quickly and discretely, accessing extended ART supplies (≥3 months) and obtaining services such as a formal transfer with ease. Privileged access thus helped participants to overcome barriers, including long clinic wait times, poor service quality, work schedules and fear of unintentional status disclosure.
  One woman said, if you have no one to help you it takes long. ‐T5, 25–34 yrs

Outreach overcame a wide variety of psychosocial and clinic‐related barriers; however, structural barriers often remained. For example, patients who reported a desire to return following outreach, but were highly mobile, only re‐engaged when they were geographically near the facility contacting them, while many personal connections linked participants to ART access more directly through supporting pick up or delivery.

(e) *Negotiating barriers through listening and counselling*: Several participants said facility outreach and personal connections provided opportunities for counselling, which helped overcome engagement barriers, including frustrations with clinic logistics and services, serostatus acceptance and depressive symptoms. Counselling included discussing challenges, processing life stressors and learning more about HIV and ART management. The counselling elements of facility outreach were crucial for a sub‐set of participants experiencing heightened vulnerability, including intimate partner violence, death of a critical member of the patient's support system, episodic or ongoing depressive symptoms and job loss or transition.

One benefit of counselling was identifying a specific barrier to re‐engagement and ways to address it. Some participants described acute fear of inadvertent status disclosure, including two whose livelihoods depended on a positive public image. Negotiating re‐engagement while maintaining anonymity was critical for their return.

Strong family and friend support served many of the same functions as facility‐associated support, such as demonstrating personal value, establishing interpersonal accountability and negotiating psychosocial and access barriers. Prominent in T2, T3 and T5, instrumental support from family members and friends included convincing ill participants to return or transporting them to the facility, and inviting and accompanying asymptomatic patients to seek care. A man from T3: Delayed linkage after testing explained that his wife was instrumental to his return.

She the one who was encouraging to go to the hospital… in fact, she was even the one who was leading the way escorting me [and also tested for HIV with me] ‐25–34 years

This type of instrumental support also sustained engagement post‐return through repeat accompanying to facility visits and support collecting medication. The mechanisms instrumental in return depended on the barriers keeping participants from care and resources (Table 1).

## 4 DISCUSSION

Narrative analysis of HIV care disengagement and re‐engagement experiences among Zambian adults identified facilitators of return to care, including a desire for physical wellbeing, encouragement, facility outreach, personal facility connections and social support. These facilitators were always interactive, with varied prominence across five care engagement patterns (trajectories). We further identified six underlying mechanisms through which the facilitators translated to re‐engagement behaviour, suggesting that while interventions to support re‐engagement may vary (e.g. facility‐led outreach vs. community‐driven social support), they should activate key underlying mechanisms to be successful (i.e. ensure a patient feels valued). Further, we found that when facilitators did not act on these mechanisms, they were unlikely to result in re‐engagement (e.g. punitive or indifferent facility outreach not conveying patient value nor improving access).

The facilitators identified added a “personal connection at the facility” to extant regional qualitative findings [27, 28, 29], and distinguished verbal encouragement as critical in all narratives, and distinct from other social support functions. These findings, complementary to primary HIV care‐seeking and retention research [27, 28, 41], represent a growing consensus that care which recognizes the whole patient yields better outcomes [42, 43].

Acknowledging that breaks from care within chronic HIV management are expected [1, 25], reasonable public health goals include more rapid return to care [13] and more efficient supportive interventions. The narratives suggest fostering patient experiences where the underlying mechanisms of return are activated for all patients prior to disengagement may support re‐engagement. Future research should investigate effective strategies to activate these mechanisms and longitudinally assess their effect on re‐engagement. Understanding the patient trajectory of care is a promising means of more efficiently targeting interventions [44, 45].

While all patients may benefit from interventions improving support and treatment access [46], narratives suggest that those with intermittent engagement (T1) may benefit most from differentiated service delivery models (DSDs) [47]. In our data, T1 was comprised of males with dynamic work requirements, preventing routine clinic attendance. Their inability to adhere to standard schedules led to disengagement, with re‐engagement catalyzed by subsequent symptomatic illness or facility outreach. While further research is needed, offering flexible DSD models to males and/or patients with dynamic work demands immediately upon ART initiation, rather than requiring them to demonstrate “stability” through prerequisite inflexible appointment attendance, may prevent treatment interruptions and, if disengagement occurs, facilitate more rapid return [48, 49].

A rich patient navigation literature suggests that navigation supports linkage to care and sustained engagement [50, 51, 52, 53]. Supported navigation of *re‐entry* through facility outreach and personal connections was a key mechanism of re‐engagement in our data. It supported those unfamiliar with re‐entry (e.g. T2 mostly engaged patients), those in T5 newly seeking ART initiation and patients with histories of negative interactions with healthcare workers (e.g. T1 intermittent engagement) or individuals with significant stigma concerns across trajectories.

For multiple participants who experienced driving and seemingly intractable barriers to re‐engagement, such as acute fear of disclosure or depressive symptoms, instrumental re‐engagement support was critical. This included counselling, shared problem‐solving and accompaniment to a health facility. Poor mental health was a prominent barrier for several participants, particularly those in T2 and T4, underlining the identified need for psychological care screening tools and referral services [54]. Active patient navigators can support problem‐solving [55, 56]. Findings also highlight the ongoing importance of families, partners and non‐medical support networks [57, 58].

The importance of personal connections to the health facility reinforces a growing call for patient‐centredness in healthcare [42, 43, 59, 60, 61], prioritizing respectful, validating patient–provider interactions. Our data and the literature consistently note the impact of health worker kindness [23, 62—67], demonstrating that positive interactions are feasible and effective. However, complex interactive factors, including overburdened systems, often result in adverse patient facility experiences [68, 69, 70]. More research is urgently needed on effective patient–provider relationship interventions [71, 72].

Patients’ self‐perception of engagement status often does not match EMR‐based characterization as lost‐to‐follow‐up [73]. This is due to differences in definitions of “in‐care” and well‐documented EMR inaccuracies regionally [31, 74, 75]. Interventions using EMR to identify patients with care gaps, such as PEPFAR Tracking and Tracing [76], may need improved linkage data [77] and/or may benefit from allowing patients to self‐describe care status. For example, tracers could ask patients to report the last facility visit or ART refill, rather than labelling “defaulters.” EMR‐based engagement research should include sensitivity analyses for potential misclassification [78, 79].

We intended for our qualitative data to help explain quantitative findings [13]. Prior quantitative analyses showed that patients contacted three or more times after missed appointments were more likely to return [13]. Our qualitative analysis elucidated that repeat contact was interpreted as health workers valuing and investing in patients. Repeat contact also increased the likelihood that outreach would come at an actionable time (e.g. when a highly mobile patient was geographically proximate to the facility). While quantitative research indicates benefit from earlier outreach [80], patients who do not return immediately may benefit from repeat, intermittent outreach. Other qualitatively identified facilitators were not quantitatively measured, suggesting that future quantitative re‐engagement research should prioritize the inclusion of measures of encouragement, social support, internal motivation and facility connections. Additionally, some quantitative factors were underrepresented or absent from the qualitative sample (e.g. rural health facility and the use of herbal remedies). We hypothesize that herbal remedy signifies efforts to resolve symptomatic illness. Future explanatory qualitative research should sample for their presence.

Our study had several limitations. It was conducted before the Zambian universal test and treat (UTT) policy. T5‐related findings may be most impacted by the switch to UTT, as T5 participants judged clinic visits not worthwhile without ART access. UTT may eliminate this concern. However, the barriers and interactive facilitators driving re‐engagement in the other four trajectories are unlikely to be significantly impacted by UTT. We did not purposefully sample to achieve trajectory saturation, as we identified patient trajectories during data analysis. Future research could explore patient characteristics and experiences within trajectories to guide intervention targeting. For example, further inquiry may reflect both male and female inclusion in T4 [81]. Reassuringly, the trajectories echo other research identifying patterns of HIV care‐seeking [44, 45] and offer rich insights into possible interventions. Our study sample was limited to Lusaka Province, a more urban, literate and wealthier area of Zambia. All participants had telephone access and were traced for participation; our findings may not be transferable to patients in different socio‐economic strata, who are more challenging to reach, or who have never experienced outreach. The median time from first re‐engagement to qualitative interview was 2.5 years (min: 1.5–max: 3.2). While our narrative approach allows for reflection and provides rich information on behavioural influences, participants who re‐engaged more recently may offer different perspectives.

## 5 CONCLUSIONS

While preliminary, the identified trajectories may guide interventions to support re‐engagement, such as targeting early, flexible ART access to those with intermittent engagement. Familiar facilitators of engagement translate to return through an underlying set of identified mechanisms that interventions must activate to achieve impact. For example, facility outreach that reminds a patient to return to care but does not affirm a patient's value or navigate re‐entry is unlikely to be effective. The demonstrated importance of positive health facility connections reinforces a growing call for patient‐centred care. Additionally, interventions should consider the important role communities play in fostering treatment motivation and overcoming practical barriers to re‐engagement and sustaining treatment.

## COMPETING INTERESTS

The authors declare no competing interests.

## AUTHORS’ CONTRIBUTIONS

LKB, SMT, EG, CEK, IS and JAD supported conceptualization. LKB, CM, CBM, SS and NM supported data collection. LKB and CM conducted formal analysis. LKB, CM, CBM, SMT, SS, KS, NM, AM, CBH, CK, IS and JAD contributed to data interpretation. LKB, JD, IS, CBH, EG and CBM acquired study funding. LKB, CBM, SMT, KS, AM, EG, CBH and IS were study investigators. LKB, CM, SMT, CK and JAD designed the methodology. SS and KS conducted project administration. LKB wrote the original manuscript draft. All co‐authors reviewed and edited the final draft.

## FUNDING

Research reported in this publication was supported by the National Institute of Mental Health of the National Institutes of Health under Award Number F31MH109378. This research was also supported by the Bill and Melinda Gates Foundation grant number OPP1105071, the Johns Hopkins University Center for AIDS Research (P30AI094189) and the Johns Hopkins Bloomberg School of Public Health Center for Qualitative Studies of Health and Medicine Dissertation Enhancement Award.

## DISCLAIMER

The content is solely the authors’ responsibility and does not necessarily represent the official views of the National Institutes of Health.

## Supporting information

TABLE S1. Participant characteristics at the time of the parent study

## Data Availability

Data access would be subject to request from the authors and review by the National Health Research Authority of Zambia.
