# Supplementary material for: Trajectories of re‐engagement: factors and mechanisms enabling patient return to HIV care in Zambia
Source: J Int AIDS Soc. 2023 Feb 24;26(2):e26067. doi: 10.1002/jia2.26067 (PMC9958345; doi:10.1002/jia2.26067)
Supplement: Supplementary file 1 — TABLE S1. Participant characteristics at the time of the parent study [file JIA2-26-e26067-s001.docx]

**Trajectories of re-engagement: Factors and mechanisms enabling patient return to HIV care in Zambia**

**Supplemental Table 1. Participant characteristics at the time of the parent study^¶^**

|  | N | |
| --- | --- | --- |
| Total Participants Enrolled | 20 | |
| *Patient Demographics and HIV treatment indicators* | | |
| **Sex** |  | |
| Female | 13 | |
| Male | 7 | |
| **Patient Chart-Documented Care Engagement Status as of December 2018** |  | |
| Active (visit Oct-Dec 2018) | | 14 |
| Re-disengaged (no visit Oct-Dec 2018)^ | 6 | |
| **Marital status** |  | |
| Never married | 7 | |
| Married | 7 | |
| Separated, Divorced, Widowed | 6 | |
| **Ever initiated ART** |  | |
| No | 13 | |
| Yes | 7 | |
| **Facility Size (number of patients)** |  | |
| Small (0 - <2,500) | 4 | |
| Medium (2,500 - <7,500) | 13 | |
| Large (7,500 – 15,000) | 3 | |
| **Interview language** |  | |
| Nyanja | 8 | |
| Bemba | 5 | |
| English | 7 | |
| **Time from incident return to qualitative interview, years** (median, IQR, min, max) | 2.5 (IQR:2.2-3.1, min:1.5, max:3.2) | |
| *Factors associated with return in quantitative analyses [13]** | | |
| **Age (years)** |  | |
| 18-24 | 2 | |
| 25-34 | 10 | |
| 35-44 | 6 | |
| 45+* | 2 | |
| **Facility Type** |  | |
| Rural Health Centre* | 0 | |
| Urban Health Centre | 16 | |
| Hospital | 4 | |
| **Wealth Tertile** |  | |
| Poorest* | 2 | |
| Middle* | 7 | |
| Richest | 9 | |
| Missing | 2 | |
| **Challenged, confronted, or educated a stigmatiser in the past 12 months** | | |
| No | 12 | |
| Once* | 4 | |
| More than once | 0 | |
| Missing | 2 | |
| **One or more > 90-day gap in EMR before parent study** | | |
| No | 11 | |
| Yes* | 9 | |
| **Contacted by facility after missed visits before study peer educator^ŧ^** | | |
| No | 16 | |
| 1-3 times | 2 | |
| >3 times* | 2 | |
| **Reported psychosocial need for return after disengagement (e.g., more encouragement, disclosure)** | | |
| No | 15 | |
| Yes* | 4 | |
| Missing | 1 | |
| **Psychosocial reason for disengagement (e.g., family conflict, depression, disclosure concern)** | | |
| No* | 16 | |
| Yes | 4 | |
| **Clinic reason for disengagement (e.g., wait too long, poor care quality)** | | |
| No* | 15 | |
| Yes | 5 | |
| **Use of herbal remedies in the past 6 months** | | |
| No | 19 | |
| Yes* | 1 | |

¶ Beres LK et al. Patterns and Predictors of Incident Return to HIV Care Among Traced, Disengaged Patients in Zambia: Analysis of a Prospective Cohort. JIAS. 2021;86(3):313-22

*attribute level more likely to return

^interviews revealed n=2 had an undocumented transfer to a different health facility

ŧ Established in the parent study that the patient had been contacted by a peer educator employed by the facility to encourage return prior to the study-employed peer educator outreach that established patient-reported disengagement and encouraged return
